# Ultra pH‐sensitive detection of total and free prostate‐specific antigen using electrochemical aptasensor based on reduced graphene oxide/gold nanoparticles emphasis on TiO_2_/carbon quantum dots as a redox probe

**DOI:** 10.1002/elsc.202000118

**Published:** 2021-08-22

**Authors:** Zahra Aayanifard, Talieh Alebrahim, Mehrab Pourmadadi, Fatemeh Yazdian, Homayoon Soleimani Dinani, Hamid Rashedi, Meisam Omidi

**Affiliations:** ^1^ School of Chemical Engineering College of Engineering University of Tehran Tehran Iran; ^2^ Protein Research Center Shahid Beheshti University Tehran Iran; ^3^ Department of Life Science Engineering Faculty of New Science and Technologies University of Tehran Tehran Iran

**Keywords:** electrochemical aptasensor, gold nanoparticle, graphene oxide, PSA detection, TiO_2_/CQD

## Abstract

The development of a rapid, sensitive, and straightforward detection method of prostate‐specific antigen (PSA) is indispensable for the early diagnosis of prostate cancer (PCa). This work relates an electrochemical method using functionalized single‐stranded DNA aptamer to diagnose PCa and benign prostate hyperplasia. The sensing platform relies on PSA recognition by aptamer/Au/GO‐nanohybrid‐modified glassy carbon electrode. Besides ferrocyanide TiO_2_/carbon quantum dots (CQDs) probe is used to investigate the effect of nanoparticle‐containing electrolyte. Optimization of incubation time of aptamer/Au/GO‐nanohybrid and volume fraction of nafion were done using Design Expert 10 software reporting 42.4 h and 0.095% V/V, respectively. In ferrocyanide medium, PSA detection as low as 3, 2.96, and 0.85 ng mL^−1^ was achieved with a dynamic range from 0.5 to 7 ng ml^−1^, in accord with clinical values, using cyclic voltammetry, square wave voltammetry, and electrochemical impedance spectroscopy, respectively. Moreover, this sensor exhibited conspicuous performance in TiO_2_/CQDs‐containing medium with different pH values of 5.4 and 8 to distinguish total PSA and free PSA, resulting in very low limit of detections, 0.028, and 0.007 ng ml^−1^, respectively. The results manifested the proposed system as a forthcoming sensor in a clinical and point of care analysis of PSA.

**Abbreviations**
AFM: atomic force microscope
AuNPs: gold nanoparticles
BPH: benign prostate hyperplasia
CQDs: carbon quantum dots
CV: cyclic voltammetry
DLS: dynamic light scattering
EIS: electrochemical impedance spectroscopy
fPSA: free PSA
FTIR: Fourier‐transform infrared spectroscopy
GCE: glassy carbon electrode
GO: graphene oxide
ILs: ionic liquids
LOD: limit of detection
PSA: PpSArostate‐specific antigen
Rct: electron transfer resistance
SEM: scanning electron microscopy
SWV: square wave voltammetry
TEM: transmission electron microscopy
tPSA: total PSA
XRD: X‐ray crystallography

## 1 INTRODUCTION

Prostate cancer (PCa) is the most common cancer and the second leading cause of cancer death and the fifth leading cause of death among men worldwide [1]. PCa usually grows very slowly, and finding and treating it before symptoms occur could improve men's health or help them live longer. Although cancer cells are often able to evade the immune system, a network of organs, tissues, and specialized cells that protects the body from infections and other conditions, not every change in the body's tissues is cancer. However, some tissue changes may develop into cancer if they are not treated. For example, the two abnormalities, hyperplasia and dysplasia, occur when cells within a tissue divide faster than expected, and extra cells build up or proliferate. Contrary to dysplasia, the cells and how the tissue is organized look normal under a microscope in hyperplasia [2, 3]. The clinical differentiation between benign prostatic hyperplasia (BPH) and PCa should be developed to guide patients to suitable treatment [4].

Prostate‐specific antigen (PSA), also named kallikrein‐related peptidase‐3, is a well‐known organ‐specific biomarker. PSA is secreted in healthy prostate tissue, BPH, and PCa of all grades and stages. In healthy adult males aged ≤50 years, the median PSA level is ∼0.6 ng ml^−1^ [5]. Total PSA (tPSA) in the blood is the combination of two different forms: free (fPSA) or in complexes with the various protease inhibitors, as proprotein or mature protein, intact or nicked. PSA levels in the blood range from <0.1 to 104 ng ml^−1^, with levels above 102 ng ml^−1^ found almost exclusively in men with advanced PCa. Since fPSA is typically less than 1.0 ng ml^−1^ in serum [6], the concentration of tPSA is considered as the basis of diagnosis in a way that a value of more than 10.0 ng ml^−1^ PCa is highly probable, and for that, less than 4.0 ng ml^−1^, the probability decreases. In the diagnostic grey zone, values between 4.0 and 10.0 ng ml^−1^, fPSA to tPSA fraction, differentiate PCa from benign BPH [7]. PSA levels in the blood are also influenced by other prostate disease conditions, such as prostatitis and age, body mass index, and race [6].

Numerous studies have been done on early diagnosis and screening of PSA, comprising fluorescence analysis [8], photoelectrochemical assay [9, 10, 11], capacitance immunoassay [12], visual immunoassay [13], aptasensor [14], electrochemical immunosensor [15, 16, 17], electrochemiluminescent immunosensor [18], field‐effect transistor [19, 20, 21], microfluidic electrochemical immunosensor [22], and surface plasmon resonance [23, 24]. The most predominant problem of all the investigations mentioned above is the application of antibodies as recognition elements. Despite their high affinity and proper selectivity, antibodies are neither time nor cost‐effective in preparation and modification [25].

Hence aptamers, allegedly artificial antibodies, in possession of high affinity and specificity, reproducibility, low price, easy synthesis and modification, inherent chemical stability, resistance to degradation, and versatility, are best‐suited to be substituted for antibodies [26, 27, 28, 29, 30, 31]. Nevertheless, despite the superiority of aptamers to antibodies, the sensitivity of aptamers to buffer conditions should be taken into account [32]. These biomolecules are single‐stranded DNA or RNA oligonucleotides, outstretched through a process names systematic evolution of ligands by exponential enrichment [33].

Graphene nanomaterials and metal nanoparticles can increase conductivity, improve specific recognition performance, and heighten biomolecule loading [31, 34, 35]. The applications of graphene oxide and reduced graphene oxide are circumfusing in many realms of science, from food and pharmaceutic industry to environmental and biomedical fields, and biosensing systems, due to the abundance of oxygen functional groups serving as a catalysis for the advancement of electrochemical biosensors, which contributes to boosting aptamer attachment and surface functionalization [31, 36, 37]. Gold nanoparticles (AuNPs) have been extensively used in diagnostics. They are not only used for signal generation through conjugation with bio‐recognition elements, antibodies, or ssDNA but also for giving rise to a considerable change in the engendered current [38]. The superiority of using AuNPs networks for surface modification to the gold electrode is the enhanced surface area, chemical approachability to the analyte, and ameliorated electrical connectivity through these networks [39, 40].

In an electrochemical cell, the movability and obtainability of charge carriers significantly affect the ionic conductivity of solvents. Erratically, ionic liquids (ILs) composed entirely of ions do not hold high conductivities compared to concentrated aqueous electrolytes, resulting from ion aggregation and formation of viscous ILs. IL‐carbon nanomaterial hybrids have lately filled the place of conventional aqueous and organic electrolytes. However, this modification technique entails some disadvantages, including high prices, inefficiency in low temperatures, and demanding electrode surface characterization and roughness control [41]. Accordingly, novel metal‐doped carbon nanomaterials shall be proposed as an electrolyte or electrode modifier. Electroactivity, photoluminescence, and low cytotoxicity of carbon quantum dots (CQDs) have engaged scientists' attention to be used in bioimaging. TiO_2_/CQDs composites have been widely utilized as photocatalysts [42, 43], yet, to the best of our knowledge, there are no reports in the literature concerning the use of TiO_2_/CQDs composites as the redox probe in the electrolyte in electrochemical aptasensor. In this investigation, TiO_2_/CQDs were prepared via a one‐step method.

**FIGURE 1:**
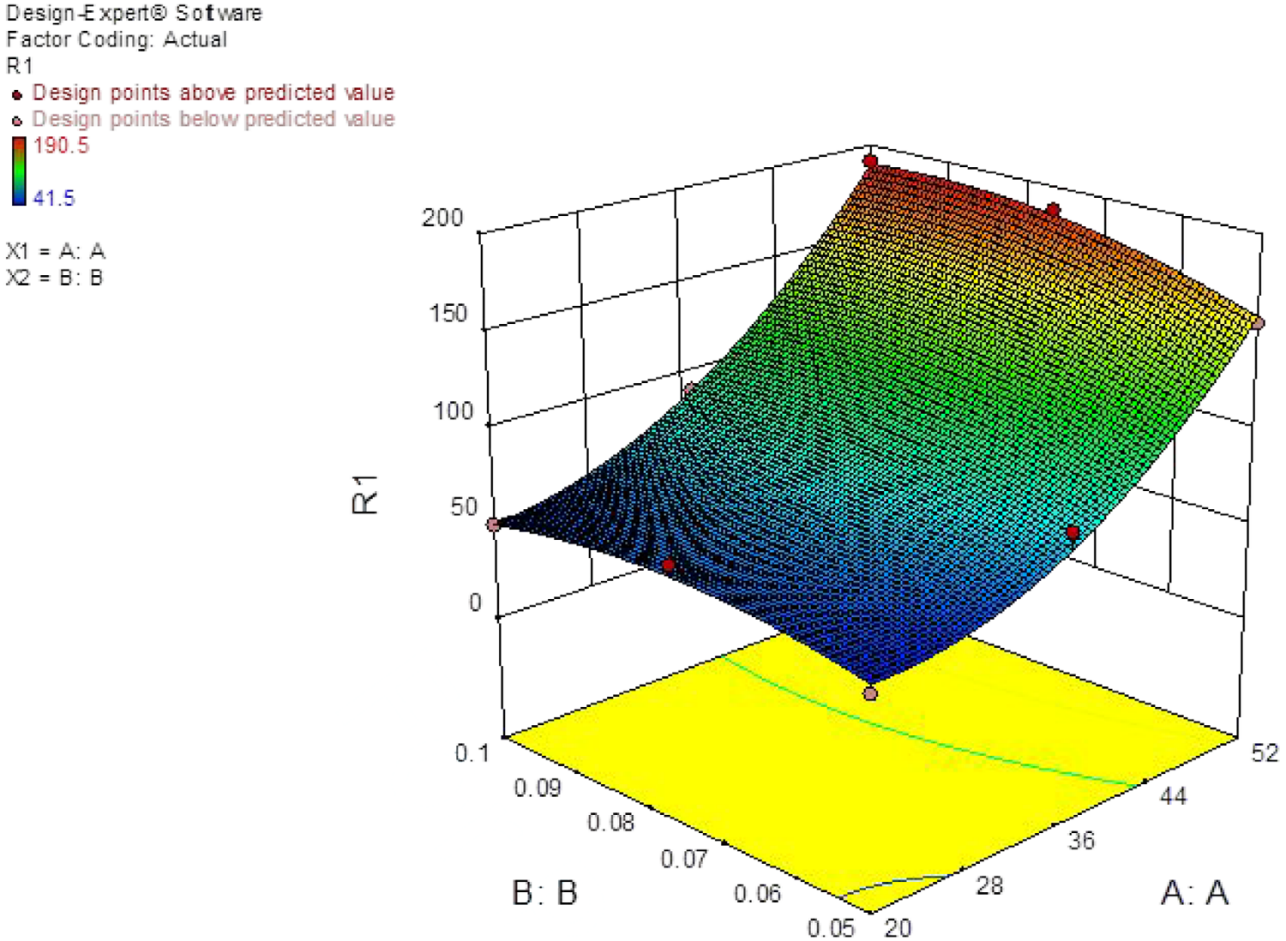
3D graph of the effect of nafion and incubation time on the current difference

Huber et al. did introduce a diagnostic tool to distinguish BPH and PCa. On the course of their experiments, PSA in the serum of patients with BPH and PCa not bound to α‐antichymotrypsin was analyzed by chromatofocusing. The procedure allowed the fractionation of the fPSA fraction into several isoenzymes. The conclusion drawn from the experiments was that the pH of the isoenzymes in sera of patients afflicted with BPH ranges from 6.6 to 7.3, while those of PCa patients were chiefly varied from 7 to 8.3 [44]. In this work, taking all the benefits of GO, AuNP, CQD, and aptamer into account, we used them to fabricate an innovative system to sense PSA selectively. A DNA aptamer, compromising a chain of 32 bases, for PSA detection has been used. A set of experiments was performed using three electrochemical techniques, cyclic voltammetry (CV), square wave voltammetry (SWV), and electrochemical impedance spectroscopy (EIS). In order to optimize nafion percentage and incubation time of the aptamer by as optimization software, Design Expert was used (Figure 1). Thereupon, the fabricated aptasensor was applied to detect PSA levels selectively.

**PRACTICAL APPLICATION**

- Ultra‐pH‐sensitive detection of total and free prostate‐specific antigen (PSA) in the range of ng ml^−1^.
- Improvement of the pH‐sensitive properties of total and free PSA for prostate cancer detection.
- A novel method to synthesis TiO_2_/carbon quantum dots as a redox probe.
- Graphene‐based nanocomposite for working electrode modification.
- The aptasensor exhibited a low response and limit of detection.

## 2 MATERIALS AND METHODS

### 2.1 Reagents and solutions

Thiolated PSA binding DNA aptamer was designed based on literature [1, 45, 46] and purchased from BioBasic (Canada, https://www.biobasic.com) with following sequence: 5′‐HS‐TTTTTTTTTTATTAAAGCTCGCCATCAAATAGCTGC‐3′ (10 OD‐HPLC). Potassium ferrocyanide trihydrate (K_4_Fe(CN)_6_.3H_2_O), hydrogen chloride (HCl), sulfuric acid (H_2_SO_4_), chloroauric acid (HAuCl_4_), sodium nitrate (NaNO_3_), graphite, potassium permanganate (KMnO_4_), and acetic acid (C_2_H_4_O_2_) were purchased from Merck (Germany). Several other materials, sodium hydroxide (NaOH), ascorbic acid (C_6_H_8_O_6_), titanium dioxide (TiO_2_), Chitosan, sodium dihydrogen phosphate dodecahydrate (NaH_2_PO_4_⋅12H_2_O), and hydrogen peroxide (H_2_O_2_), were obtained from Sigma‐Aldrich (Germany, http://www.sigmaaldrich.com). Human PSA and bovine serum albumin (BSA) were both purchased from Equitech‐Bio (USA, https://www.equitechbio.com). The aptamer was prepared, adding 299 μm sterile water to the lyophilized powder of aptamer stock and divided it into tens of 10 μl microtubes. All the tubes should be conserved at low temperatures (about −20°C) and be utilized tube by tube.

### 2.2 Apparatus

A glassy carbon working electrode (diameter = 2 mm) was ordered from Detect Co. (Tehran, Iran, http://www.detectco.com). Ag/AgCl electrode was used as the reference electrode, and platinum electrode version IRI.2000‐E was used as the counter electrode. The Ivium (vertex one) electrochemical potentiostat (Eindhoven, Netherlands, https://www.ivium.com) with the aid of Ivium software used for electrochemical measurements, including CV, SWV, and EIS. Laboratory ventilation produced by RAAD TEB NOVIN Co. (Iran, http://www.raadlabco.com) used during the aliquotation of aptamer and PSA. Centrifuge manufactured by Clement Co. (GS200 class) (Australia, https://www.clements.net.au) exploited for separation of nanosized particles. Cana way pH meter produced by SAT Co. (Iran, http://www.sat.co.ir). Ultrasonic bath class Eurosonic 4D manufactured by Euronda Co. (Italy, https://prosystem.euronda.com) used for homogenizing the nanoparticle solutions. The CV and SWV analyses were performed in the ranges of −0.5 to 0.7 V and 0 to 1 V, respectively. The EIS experiments were conducted in the frequency range between 100 kHz and 15 mHz with a perturbation amplitude of 5 mV at the potential of the oxidation peak current of CV curves.

### 2.3 Synthesis of GO

GO was synthesized from graphite powder by the modified hammers' method [47]. Briefly, 1 g of graphite powder and 0.5 g of sodium nitrate were added to concentrated sulfuric acid and stirred in a cold bath. After that, 3 g potassium permanganate was gently added to the solution, and the temperature raised to 35°C while stirring for 12 h. The obtained solution was with 500 ml of deionized water. After homogenizing the solution, 5 ml of hydrogen peroxide subjoined and washed with HCl and deionized water to remove unreacted chemicals. Graphene oxide sheets were ultimately obtained after being filtered and dried in a 40°C oven for 24 h. Twenty milliliter of GO solution with a concentration of 1 mg ml^−1^ was prepared by dissolving 20 mg of resulting GO sheets in 20 ml doubly distilled water, then placed in an ultrasonic bath in medium condition for 25 min for homogenization. Afterward, to thoroughly homogenize the solution, an ultrasonic cell with 70% intensity and 0.5 rpm speed was used for 5 min. The obtained solution was uniformly light brown.

### 2.4 Synthesis of AuNP/GO composite

In order to prepare 100 μL of chloroauric acid (0.5 M), 1.02 g chloroauric acid power was added to make a solution up to 6 ml. Afterward, 100 μl of the fully‐dissolved chloroauric acid solution was added to the mixture of 20 ml of GO solution and NaOH. The final step to synthesize a graphene oxide‐gold nanoparticle composite with a concentration of 0.25 mg ml^−1^ was to add 40 mg of arginine and insert it in a high‐temperature oven (about 130°C).

### 2.5 Synthesis of TiO_2_/CQDs

A homogeneous solution of 20 ml of ascorbic acid (0.284 M) and a solution of 0.5 g of Chitosan, and 10 ml of acetic acid (2% V/V) was prepared after being stirred on a magnetic stirrer. Subsequently, 1 g of NaOH and 0.8 g of TiO_2_ were added to the solution. After being kept in the oven (140°C) for 24 h, a solution of TiO_2_/CQDs (76.66 mg ml^−1^) was achieved [42].

### 2.6 Characterization of synthesized nanoparticles

Synthetized GO was characterized using different techniques. Surface topography and tension were surveyed by the atomic force microscope (AFM). Scanning and transmission electron microscopy (SEM and TEM) provided details about the internal composition and surface morphology. Raman spectroscopy, Fourier‐transform infrared spectroscopy (FTIR), X‐ray crystallography (XRD), and ultraviolet‐visible spectroscopy were also performed, respectively. Chemical compounds and functional groups of AuNP/GO were analyzed using FTIR. Besides, XRD and TEM were utilized to verify the loading and uniform distribution of AuNPs on GO, respectively. Afterward, in order to approve the aptamer linkage to Au/GO nano‐hybrid, the FTIR technique was performed. Parametric characterization of the TiO_2_/CQD NPs, including diameter measurement, zeta potential, and molecular weight in colloid solution, and stability of nanoparticles in suspension (pH = 5, 6, 7, 8), were presented by using dynamic light scattering (DLS) and zeta potential analysis, respectively.

### 2.7 Aptasensor's response time

**FIGURE 2:**
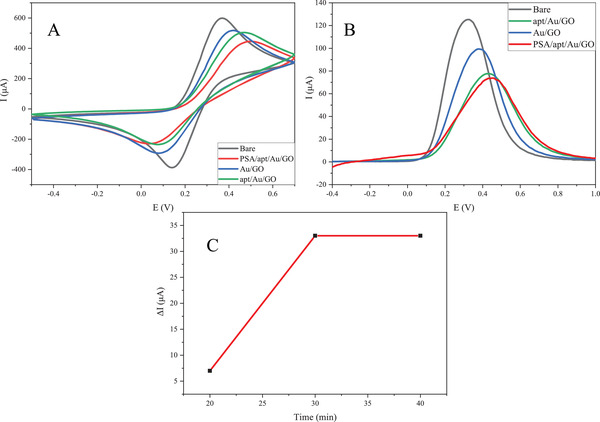
The electrochemical characterization of the nanocomposite (A) CV analysis at the scan rate of 50 mV s^−1^, (B) SWV analysis at each step of electrode modification, and (C) time profile of PSA/apt interaction based on CV techniques at different incubation time

The response time of the aptasensor was calculated as an optimization factor using the SWV technique. PSA's constant volume and concentration were coated on three similarly modified electrodes for 20, 30, and 40 min to determine the strongest conjugation of aptamer and PSA. The results demonstrated that the current difference, which is correlated to the binding strength of PSA and aptamer, increased from 20 to 30 min and merely varied after that. Accordingly, the response time of the aptasensor was fixed at 30 min. The time profile of PSA and aptamer interaction based on CV technique at different incubation times is illustrated in Figure 2C.

### 2.8 Electrochemical measurements

Prior to use, the glassy carbon electrode (GCE, 2 mm diameter) was wet polished in an eight‐shaped pattern with slurry nano‐aluminum oxide for few minutes (if the electrode has already contacted aptamer before burnishing, the time will be prolonged), followed by sequential sonication in ethanol. Then, 10 μl of apt/nafion/AuNP/GO was cautiously situated on the central area of the dried surface of the electrode under the direct light of a lamp for 30 min (attained through trial and error method) to be consummately dried. The modified electrode was rinsed with distilled water and then with 0.1 mol L^−1^ PBS (pH = 7.0) to eliminate unattached aptamer strands. After that, the electrode was fixed on an isolated chamber, and different concentrations of PSA were coated on its modified surface using a cumulative method. The bonding time of the protein was finalized on 30 min, after determining the response time of the aptasensor. Experiments revealed that 30 min is attributed to the time when the surface of the modified aptasensor is fully occupied with PSA molecules. For eliminating unattached PSA, the aptasensor was washed with 0.1 mol L^−1^ PBS (pH = 7.0). Electrochemical methods used to measure the property changes of the multilayer electrode were CV, SWV, and EIS. All electrochemical analysis was performed in a three‐electrode cell system with two different mediums. The redox probe for the first round of experiments was 1.0 mmol L^−1^ K_3_[Fe(CN)_6_]/K_4_[Fe(CN)_6_] (1:1) mixture. The scan rate and scanning potential of the CV method were 50 mV s^−1^ and between −0.5 and 0.7 V, respectively. The EIS technique was conducted in a frequency range of 10 mHz to 100 kHz with AC amplitude set in 10 mV and at a DC potential of +15 V versus Ag/AgCl, while the KCl solution was saturated. The devised Nyquist plot was fitted with appropriate equivalent circuits using the electrochemical device‐specific software. For the second round of experiments, all three electrochemical methods were similar to the previous round but with an important difference that in the second round, these sets of experiments were performed in two pH values (pH = 5.4 and pH = 8) which can be used to distinguish tPSA and fPSA concentration. All measurements were carried out using Ivium software, supported by Ivium‐Vertex1 (Netherlands, https://www.ivium.com).

## 3 RESULTS AND DISCUSSION

**FIGURE 3:**
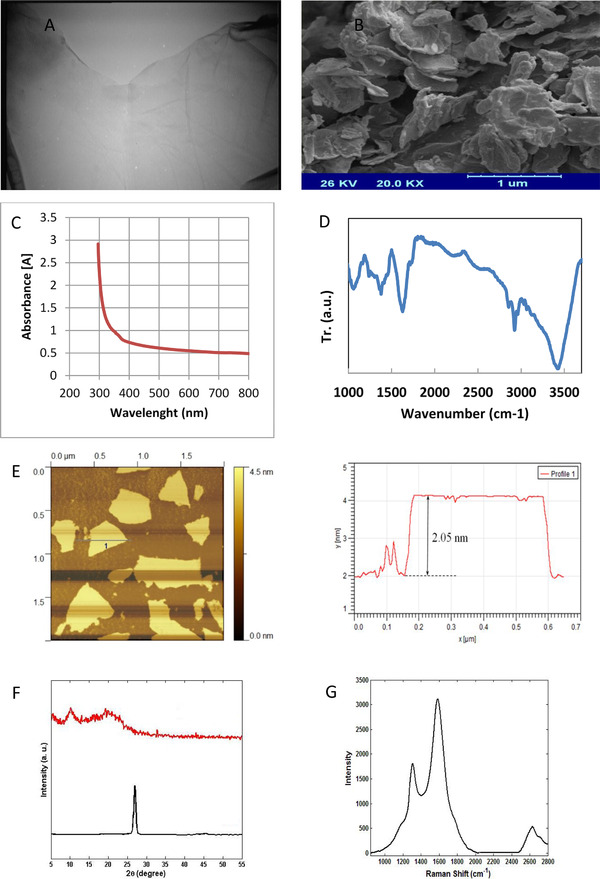
Characterization of GO nanosheets using (A) TEM, (B) SEM, (C) UV‐vis spectrum, (D) FTIR, (E) AFM, (F) XRD, and (G) RAMAN spectroscopy

Synthetized GO was characterized using different techniques. Surface topography and tension were surveyed by the AFM. Figure 3E shows a very close‐up atomic microscope image of GO. The diagram below in Figure 3E also shows the cross‐sectional profile of the GO layer specified in the figure above. As can be seen, the thickness of the GO sheet is about 2 nm, which indicates that the synthesized GO in this study is a monolayer. SEM and TEM provided details about the internal composition and surface morphology. SEM image well shows the porosity of GO sheets [46]. The SEM and TEM images of the synthesized GO are shown in Figure 3A,B, respectively. GO layers are well visible in these images, which is evidence of converting graphite plates to GO layers. Also, in Figure 3A, the monolayer of synthesized GO is clearly recognizable, which indicates the quality of synthesized GO. The presence of wrinkles is a feature of monolayer GO [48]. Raman spectroscopy, FTIR, XRD, and ultraviolet‐visible spectroscopy were also performed. As shown in the Raman spectroscopy plot in Figure 3G, peak G appears in the range of 11,600 cm, and peak D appears in the range of 11,300 cm. As a result, I_G_/I_D_ > 1 shows a significant irregularity in the aromatic structure due to the binding of the functional groups to carbon. With these interpretations, I_G_/I_D _= 1.23 value indicates a considerable irregularity in GO structure due to the connection of oxygenated functional groups. In general, it can be said that the low intensity of peak D compared to peak G indicates that the synthesized GO had few defects, and its synthesis was appropriate. Chemical compounds and functional groups of AuNPs/GO were analyzed using FTIR. Figure 3D shows GO's FTIR plot. The GO sheets synthesized in this study have specific peaks in the range of 1050 cm^−1^ (C─O bond), 1200 cm^−1^ (C─O─C bond), 1600 cm^−1^ (COOH bond), 1700 cm^−1^ (C═O bond), and broadband ranges from 3000 to 3700 cm^−1^, which belongs to the hydroxyl group (C─OH) [49]. The graph of the GO nanosystem shows that the peak around 3435 cm^−1^ can be attributed to the stretching vibration of O─H [50]. The presence of oxygenated functional groups indicates that graphite is well oxidized by oxidizing agents. In general, the peaks obtained for GO sheets are almost identical, and all fall within these ranges with a slight difference. The XRD analyses of graphite and GO are shown in Figure 3F. As shown in the upper plot, graphite has a peak at 2θ = 26°, and according to the downer plot, the peak for GO is at 2θ = 9.31°, and the distance between the layers is 9.487 Å. The distance between the GO layers is directly related to its oxidation, and with increasing oxidation, the distance between the GO layers increases [51]. According to these results, it is clear that the synthesized GO is well oxidized, and there were no impurities in the structure of graphite and GO. As shown in the UV‐visible plot of GO (Figure 3C), a protrusion or peak area of limited absorption of about 300 nm can be observed, which is related to the transfer of n to Π of the C = O bond. In addition, high absorption in the ultraviolet region (228 and 300 nm) causes the decomposition process in GO. During this process, the color of the material darkens. In addition, CO and CO_2_ are emitted. Also, keeping GO solution in light causes it to condense and accumulate. By keeping the solution in the dark, it can be stored for up to 3 years. The stability of the solution depends on the electrostatic interaction, which is attributed to the negative charge of carboxy acids and hydroxy groups. Given this, it can be concluded that in decomposition these two groups are mainly decomposed. Removal of carbon atoms causes irreversible holes in the graphene lattice, so the solution must always be kept in the dark [52].

**FIGURE 4:**
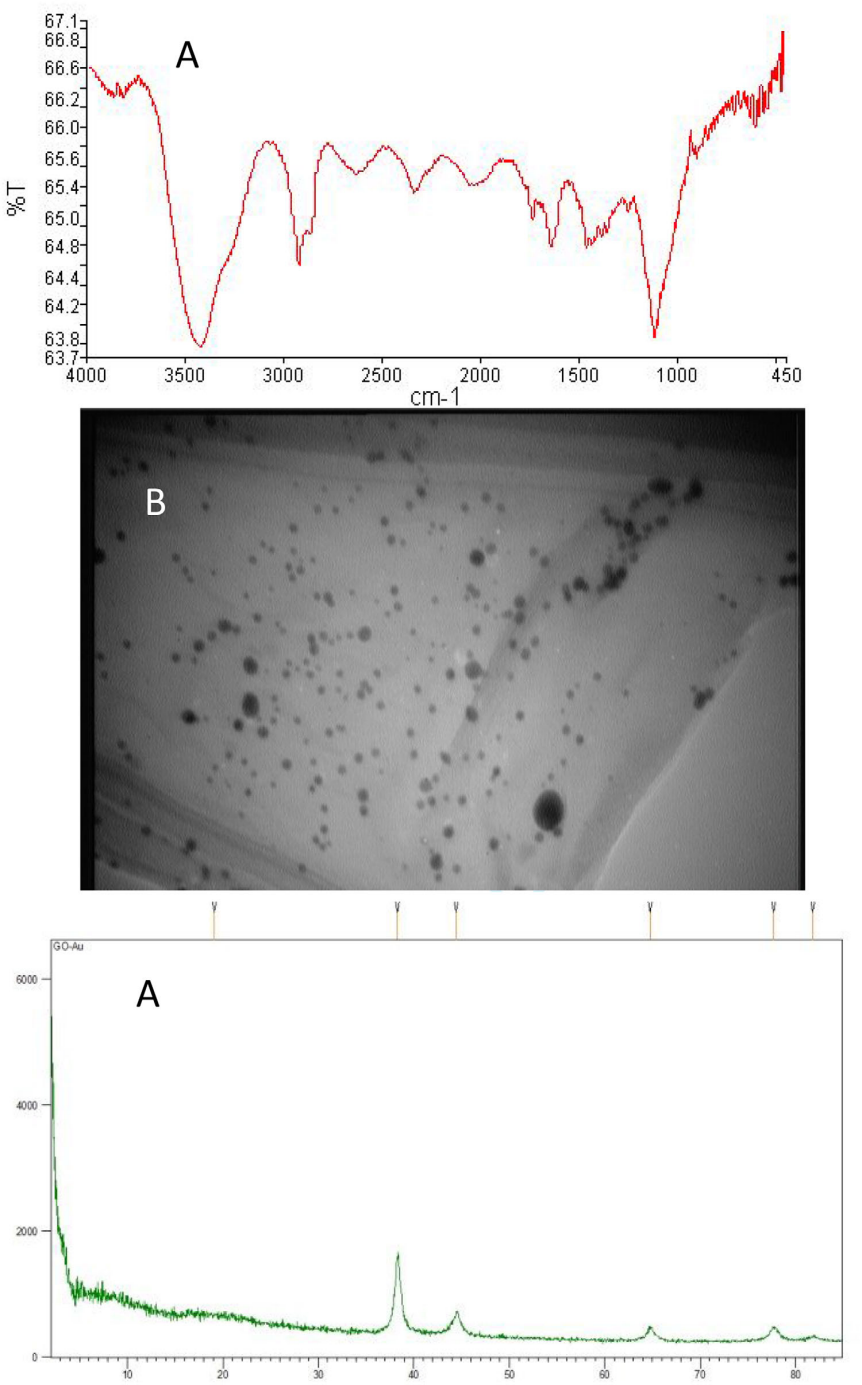
Characterization of AuNP/GO nanocomposite using (A) FTIR, (B) TEM, and (C) XRD analyses

In addition, XRD and TEM were utilized to verify the loading and uniform distribution of AuNPs on GO sheets, respectively. Figure 4A shows the FTIR of AuNP/GO nanosystems. In the AuNP/GO's FTIR spectrum, apart from the functional groups in GO, a peak at 649 cm^−1^ is observed, which is related to the Au─O─Au vibration bond. It is also observed that the peak intensity of the ‐CH_2_ bond (2923, 2853 cm^−1^) and the C═O bond (1741 cm^−1^) decreased, which may be due to the binding of gold nanoparticles to GO sheets [53]. TEM image (Figure 4B) shows the optimal sample of the AuNP/GO nanosystem. The image shows the uniform distribution of AuNPs on the surface of GO single‐layer sheets.

XRD analysis was conducted to gain detailed knowledge of the chemical composition and crystalline structure of the synthesized Au/CO nanocomposite (Figure 4C). Reduction of GO and decoration of sheets with AuNPs caused new reflection analysis in AuNP/GO's XRD patterns. In the plot related to AuNP/GO, peaks are observed in the 2θ = 44.4°, 2θ = 38.3°, 2θ = 64.7°, 2θ = 77.6°, and 2θ = 81.7°, indicating (111), (200), (220), (311), and (222) sheets [54]. As can be seen, the pattern of AuNP/GO displayed obvious diffraction peaks of AuNPs, and the peak positions and relative intensities match well with the standard XRD data for AuNP (JCPDS card, file No. 75‐0033). This confirms the formation of AuNPs on GO sheets.

**FIGURE 5:**
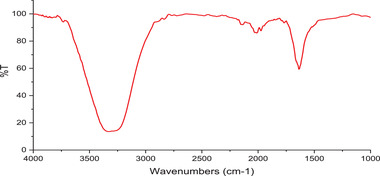
Characterization of Aptamer/GO/AuNPs using FTIR

Afterward, in order to approve the aptamer linkage to Au/GO nanohybrid, the FTIR technique was performed. As shown in Figure 5, the peak intensity of the ‐CH_2_ bond (2923, 2853 cm^−1^) has disappeared. Also, the peak with the wavelength of 1700 cm^−1^, which is related to the Au‐S bond, sufficiently indicates the binding of the aptamer to AuNP/GO.

**FIGURE 6:**
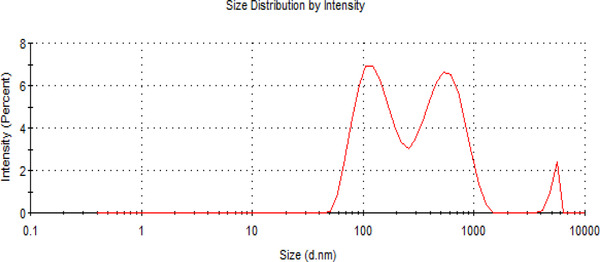
Characterization of TiO_2_/CQD NPs using DLS analysis

Parametric characterization of the TiO_2_/CQD NPs, including diameter measurement, zeta potential, and molecular weight in colloid solution, and stability of nanoparticles in suspension (pH = 5, 6, 7, 8), were presented by DLS and zeta potential analysis, respectively. The DLS analysis (Figure 6) revealed that the average diameter of TiO_2_/CQD NPs was 224 nm. Zeta analysis (Table 1) indicates that TiO_2_/CQD NPs are stable in aqueous solutions, for the average zeta potential (Z‐average) is less than −30 mV.

### 3.1 Electrochemical characterization of aptamer/gold nanoparticles/graphene oxide (apt/AuNPs/GO) modified electrode

To ensure the proper synthesis of the target nanocomposite, as well as improvements in the surface properties of the working electrode with various nanomaterial modifications, electrochemical techniques (CV, SWV, and EIS) have been used. As seen in Figure 2A,B, without any modification, the highest current peak is connected to the GCE, as it can exchange electrons without restriction with [Fe(CN)_6_]^−3/−4^. Although electron transfer should be improved by using AuNPs on the electrode surface because of the high conductivity of gold atoms, the electron exchange between the electrolyte and the electrode surface is mediated by a barrier containing oxygen atoms by the stabilization of the Au/GO nanohybrid on the electrode, making the redox response more constrained. As a consequence, the present peak decreases significantly, as seen in the blue curves. AuNPs also provide an adequate substrate to bind aptamer molecules. It raises the charge transfer resistance on the electrode surface after incubating the aptamer on the nanocomposite and thus decreasing the current amplitude (green curves). Finally, the electron transfer rate and current peak strength significantly reduce according to the red curves due to the hybridization of PSA and aptamer, hence surface deformation.

The critical parameter of PSA concentration determination after the modification process is the optimum response time. In Figure 2C, the time profile of the nanoprobe electrode in [Fe(CN)_6_]^3−/4−^ media at a constant analyte concentration is shown. A constant concentration of PSA loaded on the changed electrode surface and after 20, 30, and 40 min, respectively, in order to investigate the reaction time of the aptasensor, the CV technique was performed to obtain the current peak difference. The experiment was then conducted at a scan rate of 50 mV s^−1^ in a 2 mM [Fe(CN)_6_]^3−/4−^ medium in the potential range of −0.5 to 0.7 V. As can be shown, the analyte's hybridization is negligible at 20 min. With increasing time, the hybridization rate increased to 30 min, and it became stable in 30–40 min, suggesting that the ideal response time for this aptasensor is 30 min.

### 3.2 PSA detection in ferrocyanide electrolyte

**FIGURE 7:**
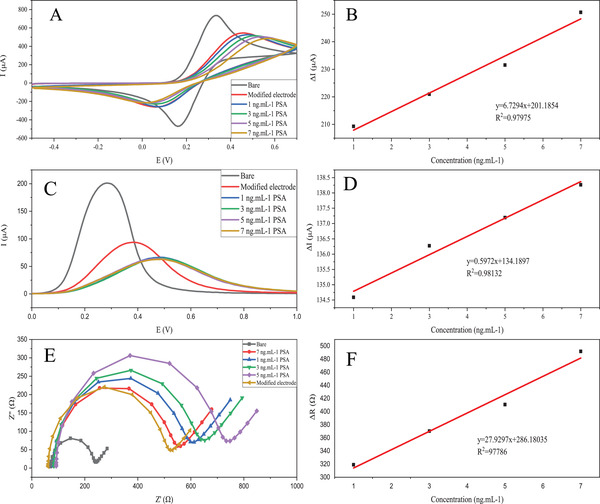
(A) CV voltammograms of apt/AuNP/GO modified electrode in [Fe (CN)6]^3−/4−^ medium at 1, 3, 5, and 7 mg ml^−1^ of PSA, (B) calibration plot derived from CV, (C) SWV voltammograms of apt/AuNP/GO modified electrode in [Fe (CN)6]^3−/4−^ medium at 1, 3, 5, and 7 mg ml^−1^ of PSA, (D) calibration plot derived from SWV, (E) Nyquist diagrams of the electrode in [Fe (CN)6]^3−/4−^ medium at 1, 3, 5, and 7 mg ml^−1^ of PSA, and (F) calibration plot derived from EIS

**FIGURE 8:**
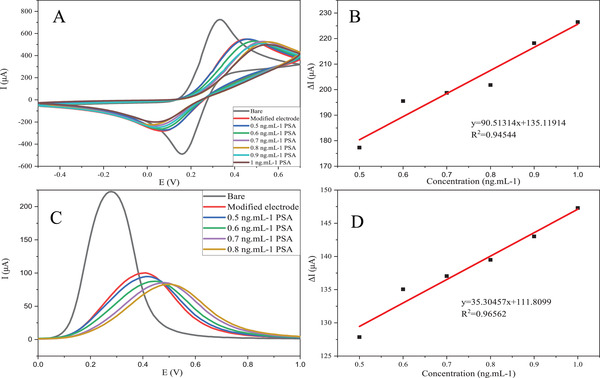
(A) CV voltammograms of apt/AuNP/GO modified electrode in [Fe (CN)6]^3−/4−^ medium at 0.5, 0.6, 0.7, 0.8, 0.9, and 1 mg ml^−1^ of PSA, (B) Calibration plot derived from CV, and (C) SWV voltammograms of apt/AuNP/GO modified electrode in [Fe (CN)6]^3−/4−^ medium at 0.5, 0.6, 0.7, and 0.8 mg ml^−1^ of PSA

Differences between maximum values of plotted curves in mentioned modes varied in direct correlation with PSA or log PSA concentration. The sensitivity of the aptasensor was measured by calculating the slope of the maximum value differences versus PSA concentration. For concentration determination in [Fe(CN)_6_]^3−/4−^ medium, initially, the base electrode signal was received by CV, SWV, and EIS tests (black curves in Figure 7A,C,E). Afterward, the stabilization of the aptamer chain was verified in the same method (red curves in Figure 7A,C,E). The range between 1 and 7 ng ml^−1^ was considered for this test. According to the results gathered in Figure 7A,C,E, the electron transfer resistance (Rct) increased at each step as the concentration increased. The current intensity has also reduced. As seen in Figure 7B,D,F, the calibration curves for CV, SWV, and EIS experiments are perfectly accurate. Furthermore, according to the formula limit of detection (LOD) = 3Sb m^−1^, where Sb is the standard deviation for blank and m is the slope of the calibration graph, the minimum amount of PSA detected by an exact value was calculated 3, 2.96, and 0.85 ng ml^−1^ in CV, SWV, and EIS, respectively, while LOQ, the minimum amount of PSA, can be quantitatively determined with accuracy and precision, was measured 0.5 ng ml^−1^. As shown in Figure 8, CV and SWV methods were repeated in a lower range of PSA concentration, and their calibration curves confirm that the aptasensor has high accuracy in these ranges (Table 2).

**TABLE 1:** Average zeta potential and conductivity of TiO_2_/CQD nanoparticles

pH	Zeta potential (mV)	Conductivity (ms cm^−1^)
5	−7.84	13.9
6	−6.38	8.16
7	−8.18	14
8	−7.81	21.3

### 3.3 PSA detection in the electrolyte containing TiO_2_/CQDs

**FIGURE 9:**
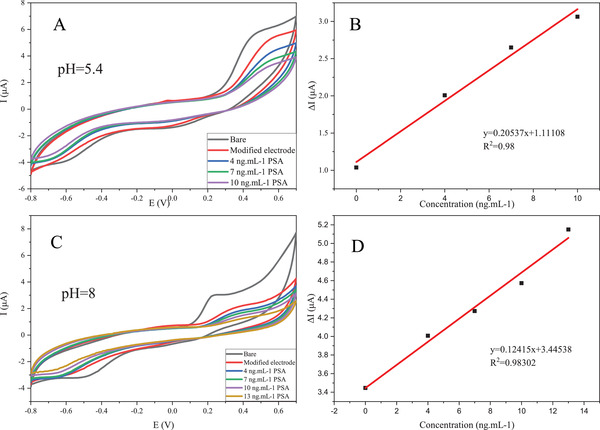
(A) CV voltammograms of apt/AuNP/GO modified electrode in TiO_2_/CQD‐containing medium at 4, 7, and 10 mg ml^−1^ of PSA in pH = 5.4, (B) calibration plot derived from CV in pH = 5.4, (C) CV voltammograms of apt/AuNP/GO modified electrode in TiO_2_/CQD‐containing medium at 4, 7, 10, and 13 mg ml^−1^ of PSA in pH = 8, and (D) calibration plot derived from CV in pH = 8

As mentioned earlier, the present aptasensor offers for the first time the ability to differentiate PCa from benign prostate hyperplasia. Different isoelectric pH values of fPSA, 6.6–7.3 for BPH and 7–8.3 for PCa, were the differentiating factor.  The difference between isoelectric points is reported to be related to the irregular glycosylation process in the dysplastic cells of the prostate. Using this concept as a diagnostic tool, we were able to measure tPSA and fPSA separately by changing the medium's pH value. The nanoprobe electrode in the TiO_2_/CQD medium was used for this purpose. CV experiments have been used to examine changes in current intensity and potential changes in two different pH values, the outcomes of which can be seen in Figure 9A,C. As seen in these two figures, as the concentration of the sample solution increases, the resistance of the electron transfer on the surface of the electrode increases as the surface configuration changes, and the active sites are blocked, resulting in a decrease in the peak current of the graph. The calibration curves for the CV tests are also plotted to determine the linear range of the aptasensor and to calculate R^2^ in Figure 9B,D. According to the aforementioned formula, the LODs in pH = 5.4 and pH = 8 are obtained 0.028 and 0.007 ng ml^−1^, respectively (Table 3).

**TABLE 2:** Results from the design of experiments based on CCD

Nafion (% V/V)	Incubation time of the aptamer (h)	Current (μA)
0.050	20	41.5
0.050	52	154.9
0.100	20	49.6
0.100	52	190.5
0.075	20	65.2
0.075	52	187.5
0.050	36	81.6
0.075	36	85.0
0.100	36	90.2

**TABLE 3:** Analysis of variance related to the volume percentage of nafion and incubation time

Parameters	SSE	Degree of freedom	MSE	F‐value	*P*‐value
Sample	26246.08	5	5249.22	74.15	0.0024
Incubation time (A)	23637.93	1	23637.93	333.92	0.0004
Nafion % (B)	455.88	1	455.88	6.44	0.0848
AB	189.06	1	189.06	2.67	0.2000
A^2^	1713.08	1	1713.08	24.2	0.0161
B^2^	250.13	1	1412.67	250.13	0.1567
		R^2^	99.2%		

### 3.4 Selectivity

**FIGURE 10:**
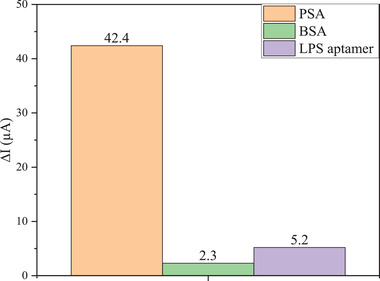
Comparison of nanoprobe electrode signal response for PSA, BSA, and LPS aptamer

The selectivity is a determining factor in the application of the aptasensor in real samples. This assay was carried out by detecting the CV current changes in the presence of PSA (2.5 ng ml^−1^), BSA (5 ng ml^−1^), and lipopolysaccharide (LPS) aptamer (100 μM) with 6′NH_2_ modification with the following sequence: (CTTCTGCCCGCCTCCTTCCTAGCCGGATCGCGCTGGCCAGATGATATAAAGGGTCAGCCCCCCAGGAGACGAGATAGGCGGACACTOD5 HPLC 5′ Mod Amino Modifier—NH_2_ C6) [40]. The current deviation of BSA and LPS aptamer modified electrode from the electrode without biomolecule load were 5.8% and 11.7%, respectively. These results are representative of the high selectivity of the aptasensor (Figure 10).

## 4 CONCLUDING REMARKS

This investigation eventuated in developing an electrochemical aptasensor for the diagnosis of PCa and BPH. Nanoparticles were mainly utilized to fabricate the aptasensor; GO nanosheets as proper nanocarriers of aptamer, AuNPs as a thiolated‐aptamer modifier TiO_2_/CQD nanoparticles in ion transfer facilitation in the electrochemical cell. The electrochemical cell was first comprised of ferrocyanide and then TiO_2_/CQDs‐containing electrolyte. To the best of our knowledge, TiO_2_/CQD NPs have been used in electrolyte modification for the first time. The use of thiolated aptamer results in cost reduction by using a GCE instead of a gold electrode. In an optimized condition, the concentration range of PSA detection was 0.5–7 ng ml^−1^, with an LOD of 0.007 ng ml^−1^ by the engineered aptasensor. Two different pH = 5.4 and 8 were also specified to demarcate PCa and BPH. The aptasensor exhibited acceptable selectivity, and sensitivity to a high degree and good duplicability. Consequently, this cost‐effective, ultrasensitive, and selective PSA aptasensor can be taken as a promising practical approach for PSA detection and also PCa differentiation from BPH.

## CONFLICT OF INTEREST

The authors have declared no conflict of interest.

## Supporting information

Supporting Information

## Data Availability

The data that support the findings of this study are available from the corresponding author upon reasonable request.
