# Supplementary material for: Ultra pH‐sensitive detection of total and free prostate‐specific antigen using electrochemical aptasensor based on reduced graphene oxide/gold nanoparticles emphasis on TiO2/carbon quantum dots as a redox probe
Source: Eng Life Sci. 2021 Aug 22;21(11):739–52. doi: 10.1002/elsc.202000118 (PMC8576073; doi:10.1002/elsc.202000118)
Supplement: Supplementary file 1 — Supporting Information [file ELSC-21-739-s001.pdf]

Preparation of aptamer/AuNP/GO

In each set of experiments, 100ul of aptamer graphene oxide gold nanoparticle was required. Accordingly, aptamer and Au/GO (1:49) were mixed and incubated in the refrigerator temperature (-4 °C) for three different spans of time (20, 36, and 52 h). During this time, the aptamer became bonded with gold nanoparticles by strong covalent bonds between -SH and gold atoms, as displayed in Fig.2. Subsequently, three different %V/V nafion (0.05, 0.075, and 0.1) was added to the incubated aptamer (1:4) for 15mins; this step was performed on the surface of the electrode.

Optimization of volume percentage of nafion and incubation time

The conditions of biosensors are commonly optimized using trial and error methods [8, 10], while it is also possible to improve the efficiency of the aptasensors through the design of experiments [26]. In this study, to optimize two parameters, the volume percentage of nafion and incubation time, design of examination (DOE) with the help of response surface method (RSM) was employed via Design Expert Software version 10 [26]. These two variables have marked effects on the operation, stability, and selectivity of the aptasensors. The experiments were designed using a central composite design (CCD). The difference between the maximum current of CV in the presence and absence of Au/GO nanosystem was analyzed as proof of the proper incubation process. Variance analysis for the current difference can be seen in Table 1. In this analysis, parameters in possession of P-values lower than 0.05 plus confidence level upper than 0.95 were meaningful [28]; thus, the rest of the parameters were omitted. The optimized values, 0.095 %V/V and 42.4 h were remained constant in subsequent experiments.

Figure 1 shows the 3D image of Nafion concentration and time effect on the difference in electric current.

Optimal TiO_2/CQD concentration

Continuous volumes of TiO_2/CQD at a concentration of 76.66 ng.mL^{-1} were gradually injected into 50 mL of PBS, and each time a CV test was performed. Starting with a volume of 400 µL and increasing to a volume of 2400 µL continuously, the amount of TiO_2/CQD in PBS was increased. Thus, due to

the clearer peak of CV oxidation at a concentration of 36.797 ng.mL^{-1}, TiO_2/CQD nanoparticles were used at this concentration.

Preparation of TiO_2/CQDs buffer

In order to prepare buffer solutions with pH values in the range of 5.8 to 8, different portions of Na_2HSO_4 and NaH_2SO_4 were added to PBS (pH=7). Distilled water was added to 1.92 mL of TiO_2/CQDs (76.66 mg.mL^{-1}, pH=6) to the solution up to 4 mL, to obtain an optimized solution of TiO_2/CQDs (37.66 mg mL^{-1}). The same process was redone, but at different pH value (pH=8). These two solutions, unlike previously prepared solutions, were directly used as the medium.
